# Unexpected mitochondrial lineage diversity within the genus *Alonella* Sars, 1862 (Crustacea: Cladocera) across the Northern Hemisphere

**DOI:** 10.7717/peerj.10804

**Published:** 2021-02-01

**Authors:** Anna N. Neretina, Dmitry P. Karabanov, Veronika Sacherova, Alexey A. Kotov

**Affiliations:** 1 A.N. Severtsov Institute of Ecology and Evolution, Russian Academy of Sciences, Moscow, Russia; 2 I.D. Papanin Institute for Biology of Inland Waters, Borok, Yaroslavl State, Russia; 3 Charles University Prague, Prague, Czech Republic

**Keywords:** Cladocera, Phylogeography, Genetics, Biodiversity, Biogeography

## Abstract

Representatives of the genus *Alonella* Sars (Crustacea: Cladocera: Chydorinae) belong to the smallest known water fleas. Although species of *Alonella* are widely distributed and often abundant in acidic and mountain water bodies, their diversity is poorly studied. Morphological and genetic approaches have been complicated by the minute size of these microcrustaceans. As a result, taxonomists have avoided revising these species. Here, we present genetic data on *Alonella* species diversity across the Northern Hemisphere with particular attention to the *A. excisa* species complex. We analyzed 82 16S rRNA sequences (all newly obtained), and 78 COI sequences (39 were newly obtained). The results revealed at least twelve divergent phylogenetic lineages, possible cryptic species, of *Alonella*, with different distribution patterns. As expected, the potential species diversity of this genus is significantly higher than traditionally accepted. The *A. excisa* complex is represented by nine divergent clades in the Northern Hemisphere, some of them have relatively broad distribution ranges and others are more locally distributed. Our results provide a genetic background for subsequent morphological analyses, formal descriptions of *Alonella* species and detailed phylogeographical studies.

## Introduction

Water fleas (Crustacea: Cladocera) are microscopic crustaceans common to continental waters (*Kotov, 2013*). *Daphnia* O.F. Müller is familiar to the public as a study subject in classrooms and as a food source in the aquarium industry. But related cladocerans that are crucial elements in littoral and benthic ecosystems are mostly unknown to the public. A rough estimate of the approximate number of cladoceran individuals in the World, based on the total area of inland waters being 10^6^ km^2^ and an underestimated average number of cladocerans being 1,000 individuals per 1 m^2^, gives 10^15^ individuals (*Smirnov & Kotov, 2010*). Most of these cladocerans do not belong to the genus *Daphnia*, but their ecological importance is immense and very little is known about their diversity.

While *Daphnia* is universally accepted as an important model for ecological, toxicological and genetic studies (*Lampert, 2011*), we still know very little about other water fleas. However, in the last ten years, substantial progress has been made by integrative taxonomic and phylogenetic studies of non-model species groups from the families Daphniidae (*Ishida, Kotov & Taylor, 2006; Petrusek et al., 2008; Dlouhá et al., 2010; Kotov & Taylor, 2010; Popova et al., 2016; Bekker et al., 2018; Karabanov et al., 2018; Kotov & Taylor, 2019; Kotov et al., 2020*), Bosminidae (*Kotov, Ishida & Taylor, 2009; Faustova et al., 2011; Faustova, 2017*), Eurycercidae (*Bekker, Kotov & Taylor, 2012*), Moinidae (Petrusek, Černy & Audenaert, 2004; Bekker et al., 2016; Montoliu-Elena, Elías-Gutiérrez & Silva-Briano, 2019; Ni et al., 2019), Chydoridae (*Sacherová & Hebert, 2003; Belyaeva & Taylor, 2009; Kotov et al., 2016; Sinev, Karabanov & Kotov, 2020*), Polyphemidae (*Xu et al., 2009*) and Leptodoridae (*Xu et al., 2011*). Based on these works, water fleas are no longer considered as exemplars for cosmopolitanism (Frey 1982; Frey 1987). There has also been significant progress in large-scale biogeographic reconstructions for these tiny animals. But the slogan “everything is everywhere” (Baas Becking, 1934) still may be applied to the biogeography of taxonomically difficult groups of cladocerans, such as the genus *Alonella* Sars (Anomopoda: Chydoridae: Chydorinae). This genus includes the smallest representatives of the water fleas known to date. The adult specimens of *Alonella* do not exceed 0.45 mm in length and are barely visible to the naked eye (*Smirnov, 1971; Smirnov, 1996*). Although *Alonella* is widely distributed all around the World and often abundant in acidic and mountain water bodies (*Smirnov, 1996; Van Damme & Eggermont, 2011*), its diversity is still poorly studied. Morphological and genetic study of *Alonella* is made difficult by the small body size. Although some other small-bodied cladoceran taxa are intensively studied now, taxonomists have avoided revision of the species of *Alonella*. Since N.N. Smirnov’s monographs on the chydorids (*Smirnov, 1971; Smirnov, 1996*), only a single new species of *Alonella* has been described (*Alonso & Kotov, 2017*). Also, few attempts to isolate and sequence DNA have been carried out for this genus mainly due to the molecular barcoding efforts (Costa et al., 2007; Jeffery, Elías-Gutiérrez & Adamowicz, 2011; Prosser, Martínez-Arce & Elías-Gutiérrez, 2013), and studies of the chydorid generic relationships (*Sacherová & Hebert, 2003)*.

**Figure 1:**
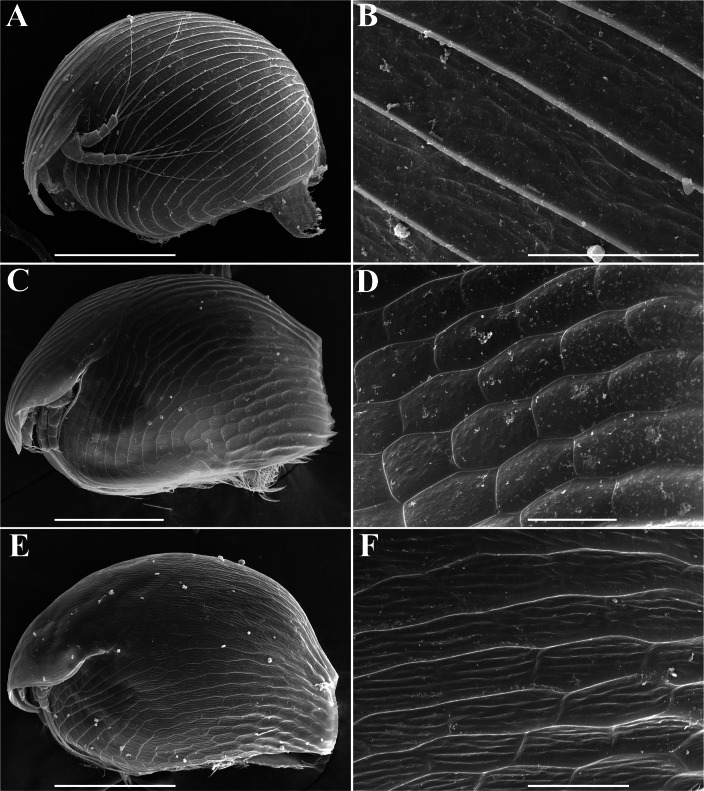
*Alonella* parthenogenetic females identified based on morphological characters. General view and sculpture of valves of *A. nana* (A–B) (from Lake Glubokoe, Moscow Area, Russia); *A. exigua* (C–D) (from Meertvoe Lake in the vicinities of Krasnaya Pahra village, Moscow Area, Russia); *A. excisa* (E–F) (from the roadside mire, Yakutia Republic, Russia). Scale bars: 0.1 mm for A, C, E; 0.02 mm for B, D, F.

In fact, only three morphospecies of *Alonella*: *A. nana* (Baird), *A. exigua* (Lilljeborg) and *A. excisa* (Fischer) (Figs. 1A–1F) are recognizable now in the Northern Hemisphere following the Smirnov’s key (*Smirnov, 1996*). *A. nana* is characterized by a sub-globular body shape and prominent diagonal lines on the valves (*Smirnov, 1971; Hudec, 2010*) (Figs. 1A–1B). *A. excisa* and *A. exigua* have an elongated body and polygonal ornamentation (Figs. 1C–1F). There are small dots within each polygon in *A. exigua* (Figs. 1C–1D), while each polygon in *A. excisa* carries short parallel striation (Figs. 1E–1F). Each of these three morphospecies has a very wide geographic range. As such, they are candidates for groups of sibling species (*Frey, 1982; Frey, 1987*). Indeed, preliminary morphological evidence suggested the existence of several species within the *A. excisa* complex (*Kotov et al., 2013*). The status of other *Alonella* and *Alonella*-like taxa (out of *nana*, *excisa* and *exigua* groups) (*Smirnov, 1971; Smirnov, 1996*) remains unclear. The aim of the present work is to elucidate the mitochondrial lineage diversity and preliminary biogeography of *Alonella* (especially of the *A. excisa* species complex) across the Northern Hemisphere using different methods of the OUT delimitation. The evidence is based on variation in mitochondrial 16S ribosomal RNA (16S) and cytochrome c oxidase subunit I (COI) genes.

## Material and Methods

### Ethics statement

Field collection in public property in Russia does not require permissions. Sampling in the state natural reserves of Russia was conducted with special verbal permission of their heads (O.P. Elizarova, Pinezhsky State Natural Reserve; T.I. Shpilenok, Kronotsky Biosphere Reserve; Y.P. Sushitsky, Khanka Nature Reserve). Ethiopian samples were collected in the frame of the Joint Ethio-Russian Biological Expedition, with permission of Ministry of Environment, Forest and Climate Change of Ethiopia. Samples in Mongolia were taken in the frame of the Joint Russian-Mongolian Complex Biological Expedition with special permission of the Ministry of Nature, Environment and Tourism of Mongolia. Samples in South Korea were collected in the frame of cooperation between A.A. Kotov and the National Institute of Biological Resources of Korea and does not require special permission. All the localities in Ethiopia, Mongolia and South Korea belong to public property. They are relatively large lakes, streams, affluents of rivers or roadside ponds.

### Field collection, identification and photographing

Samples were collected by plankton nets (with mesh size of 30–50 µm) of different construction and fixed immediately after sampling in 96% ethanol. All samples were preliminarily inspected in the laboratory under a binocular stereoscopic microscope LOMO (Open Joint-Stock Company, Russia). In samples where *Alonella* taxa were detected, the whole volume of sample was examined under light microscope Olympus BX41 for accurate identification based on morphological characters via standard keys (*Smirnov, 1971; Smirnov, 1996*). Selected individuals were studied under a scanning electron microscope CamScan MV 2300 (Tescan, Czech Republic) as described previously (*Kotov, 2013; Neretina & Kotov, 2015*).

### DNA sequencing

One to three parthenogenetic females from each population (see Table S1) predominantly of the *A. excisa* species complex were studied for genetic profiles. Identification of each parthenogenetic female used in the genetic analysis was especially re-checked under a stereoscopic microscope in order to avoid possible mistakes related with situations when a sample contained several *Alonella* species simultaneously. Selected individuals were placed into 96-well PCR plates and air-dried. DNA from individual crustaceans was extracted in 50 µl of proteinase K solution, according to the protocol of Schwenk et al. (1998)). PCR reactions were carried out in 25 µL volume, containing 5 µL of genomic DNA, 6.2 µL of distilled H_2_O, 0.65 µL (10 µM) of each primer to amplify either COI (COI-F: 5′-TGTAAAACGACGGCCAGTTCTASWAATCATAARGATATTGG-3′; COI -R: 5′-CAGGAAACAGCTATGACTTCAGGRTGRCCRAARAATCA- 3′) or 16S (16S-F: 5′-CCGGAATTCCGCCTGTTTATCAAAAACA-3′; 16S-R: 5′-CCCAAGCTTCTCCGGTTTGAACTCAGAT-3′) (see details on COI primers in Prosser, Martínez-Arce & Elías-Gutiérrez (2013) and details on 16S primers in Simon et al. (1994)) and 12.5 µL of PPP Master Mix (Top-Bio, the Czech Republic) in a thermocycler GeneTouch (Hangzhou Bioer Technology Co., China). The PCR cycles both for COI and 16S fragments included the following steps: initial denaturation at 92 °C for 3 min, 40 cycles (denaturation at 94 °C for 1 min, annealing at 50 °C for 1 min, and elongation at 72 °C for 1.5 min), and final elongation at 72 °C for 5 min. Amplified PCR products were sequenced using forward and reversed primers or only via forward primers. In the first case, a single consensus sequence was assembled using the forward and reverse sequences using CodonCode Aligner v. 6.0.2 (CodonCode Corp, USA) and checked for possible stop-codon presence. DNA sequences were submitted to the NCBI GenBank database (accession numbers MN608113 –MN608151 for COI and MN598677 –MN598759 for 16S) (Table S1).

### Analysis of sequences and reconstruction of phylogeny

The authenticity of all newly obtained sequences was verified by BLAST comparisons (*Boratyn et al., 2013*). We also added two species of Chydoridae (*Alona affinis* (Leydig, 1860) and *A. setulosa* Megard, 1967) as outgroups and existing sequences of *Alonella* from GenBank (Table S1) to our study. The sequences were aligned via a software package MAFFT v.7 (Katoh & Standley, 2016) on the server of the Computational Biology Research Center, Japan (http://mafft.cbrc.jp). For alignment of the protein coding COI locus we used the “Translation Align” option with strategy FFT-NS-i. For the 16S locus, we used the Q-INS-i algorithm, which considers secondary structure. Searching of appropriate models and partitioning schemes were fulfilled via ModelFinder v.1.6.9 (*Kalyaanamoorthy et al., 2017*) on the web-service of the Center for Integrative Bioinformatics Vienna, Austria (http://www.iqtree.org). Selection of the best model was carried out based on the best minimal values of the Bayesian information criterion (BIC) (*Posada & Buckley, 2004*). Parameters of nucleotide substitutions were identified in ModelFinder (*Kalyaanamoorthy et al., 2017*) as K3Pu+F+G4 for 16S, and for COI triplets: 1st –TN+F+I, 2nd –TVM+F+I and 3rd –HKY+F+G4. Parameters of the model BIC were almost identical to those found via the corrected Akaike’s information criterion (AICc) (Posada & Crandall, 2001).

Phylogeny reconstruction was carried out for each locus separately. Also, we reconstructed a joint consensus tree based on the whole set of unlinked data using the maximum likelihood (ML) or Bayesian (BI) methods. For ML analysis we used an algorithm IQ-TREE v.1.6.9 (*Nguyen et al., 2015*), as implemented on the CIBIV web-server (*Trifinopoulos et al., 2016*). Each set of sequences was analyzed based on the best model, which was automatically calculated by W-IQ-TREE (*Trifinopoulos et al., 2016*). As the branch supporting test, we used 1k replics in UFbootstrap2, requiring significantly smaller computational resources as compared with traditional supporting tests and demonstrating a higher effectiveness of such calculations (*Hoang et al., 2018*). When conducting BI, the posterior probabilities (*Bolstad, 2007*) were calculated in BEAST2 v.2.6 (Bouckaert et al., 2019). All parameters of substitution models were identified for BI-trees via BEAUti (*Drummond et al., 2012*) (part of BEAST2 package) using the BIC criterion. In each BEAST2-analysis, we conducted four independent runs of MCMC (100M generations, with selection of each 10k generation) with effectiveness control in “R We There Yet” (RWTY) for “R” statistical language (*Warren, Geneva & Lanfear, 2017*). A consensus tree based on the maximum clade credibility (MCC) was obtained in TreeAnnotator v.2.6 (*Drummond et al., 2012*) with half increased burn-in rate determined in RWTY (but no less than 20%).

ML-testing MEGA-X (*Kumar et al., 2018*) rejected a strict molecular clock. Therefore, we used an uncorrected relaxed clock with lognormal distribution, allowing to vary the substitution speed in different portions of the tree (Drummond et al., 2006). Speciation was analyzed using the Yule process approximation (*Steel & McKenzie, 2001*). *Alona* sequences from the GenBank are used as *a priori* designated outgroup. Having no additional information on stationary frequencies and parameters in nucleotides substitution, we ignored the priors of Dirichlet’s distribution due to their weak positive influence on the phylogeny reconstructions (*Sarver et al., 2019*). After conclusion on the full consensus in the main clades between BI and ML, we represented in our illustrations only ultra-metric BI trees, with branches supports for key nodes only.

Correlation between phylogenies based on different genetic loci is a special issue in every phylogenetic reconstruction (*Nei & Kumar, 2000*). We carried out a comparison between trees made in BEAST2 separately for 16S and COI, analyzing sequences exactly from the same vouchers on the tanglegram constructed in Dendroscope-3 (*Scornavacca, Zickmann & Huson, 2011*). General topologies of reconstructed 16S and COI trees were similar, which allowed us to analyze not only individual gene phylogenies, but also to use a more powerful coalescent methods to analyze the relationship between the reconstructed trees through the calculation of a multigenic supermatrix (BEAST2 (*Bouckaert et al., 2019*)) and by merging individual gene trees (ASTRAL-III (*Zhang et al., 2018*)). No fundamental differences of both tree topologies were found. We deleted branches with low support (*Zhang et al., 2018*). However, this transformation failed to improve the output tree. Thus, we have a justification for our reconstruction of multilocus phylogeny and combination of data even in the presence of “gaps” (*Molloy & Warnow, 2018*) as we did not have sequences of both genes from all specimens.

### Species delimitation

There is no universal approach for species delimitation based on the OTUs in the gene sequence-based trees (*Kartavtsev, 2018*), and we used three most common approaches to the OTUs delimitation based on single locus data, as well as the whole dataset. Since a preliminary prior data sorting on the possible OTUs is required for most computer packages, we conducted an analysis of the tree reconstruction for each locus separately based on the ABGD distance method, coalescence models in the ‘splits’ and ‘bGMYC’ packets, as well as through the Poisson analysis of mPTP.

The simplest method based on analysis of a threshold of divergence, Automatic Barcode Gap Discovery (ABGD) (Puillandre et al., 2012), was realized based on the web-server Atelier de BioInformatique, France (http://wwwabi.snv.jussieu.fr/public/abgd/abgdweb.html). Single values for both mitochondrial loci were selected by us: Pmin = 0.001, Pmax = 0.1, Steps = 100, *X* = 10, Nb = 25.

The second method, applying the coalescence approach based on general mixed Yule-coalescent model (GMYC) (*Pons et al., 2006*) with the “classic” implementation of GMYC, was performed in the ‘splits’ package (*Fujisawa & Barraclough, 2013*) for Microsoft “R-Open & MKL” software v.3.5.3 x64 (http://mran.microsoft.com/). As the input tree, we used an ultrametric BI-tree made in BEAST2 for each locus. As it is known that realization of GMYC in the case of a complicated structure of natural populations leads to considerable over-estimation of the number of recognizable taxonomic units (Lohse, 2009), we used Bayesian realization of the general mixed Yale model and coalescence in order to increase (at least partly) the reliability of GMYC conclusions (*Reid & Carstens, 2012*) in the package ‘bGMYC’ for “R”. Input trees for ‘bGMYC’ were the same as for the ‘splits’ analysis. Sorting, re-rooting of the trees and removing the outgroups was carried out via the “R” package according to the script of Sweet et al. (2018). We randomly selected 100 ultrametric trees from the primary set to the ‘bGMYC’ processing with the following parameters: 100 k MCMC generations with 50% annealing; the range of threshold values from 2 to the maximum number of sequences in the data set; start values for both Yale and coalescence models according to Reid & Carstens (2012) as the most usable for the majority datasets. These sets allowed us to obtain the distribution of “Coalescence to Yule”>>0, what is a sign of a good fit of the model parameters to the data. The threshold level of the cladogenesis reliability was accepted as *P* > 0.95 and *P* > 0.99, which allows us to reduce the probability of an excessive lumping in the taxonomic structure.

The third method of the species delimitation was as the previous one, but based on the Poisson tree processes (PTP). This approach aims to distinguish speciation processes among the species from diversification processes within the species, but it analyzes the number of substitutions between branching events instead of time intervals. For data processing, we used multi-rate Poisson Tree Processes, mPTP (*Kapli et al., 2017*) on the web-server of Heidelberg Institute for Theoretical Studies (http://mptp.h-its.org/). As the input tree, we used the phylogenetic ML-tree obtained used W-IQ-TREE for each locus.

A new version of “tr2” (*Fujisawa, Aswad & Barraclough, 2016*) for Python v.3.7 x64 (http://www.python.org) was used as a method for multi-locus taxonomy. This method is based on the identification of a transition point between species branching and branching within species via estimation of observed and expected levels of genes congruence according to the coalescence theory for rooted triplets topologies. We used an option of testing of the OTUs *apriori* partitioning on the consensus ultrametric tree in BEAST2 for both loci unlinked. However, this mechanistic approach does not allow to adjust the model taking into account the genetic features and biology of different groups of organisms. As an alternative method, we used a Bayesian approach for the delimitation of multi-species coalescence model using molecular sequences from multiple loci in STACEY v.1.2.4 (*Jones, 2017*) for BEAST2. In fact, this method is a version of the multi-species coalescence model used in *BEAST (Heled & Drummond, 2009). There the birth-death-collapse model is used in order to estimate the species tree (*Jones, 2017*). Final phylogenetic relationships were estimated via STACEY in four independent runs for the whole data set. Each run consisted of 50M MCMC generations, with selection of every 10k with 10% pre-annealing. STACEY log files were examined in Tracer v.1.7.1 (*Rambaut et al., 2018*) to assess whether the runs have reached the stationary phase and converge on model parameters (ESS > 400). Support of topologies was evaluated in STACEY by constructing a tree of maximum reliability in TreeAnnotator (part of BEAST2 package) after rejection of a half of all estimated trees. Species delineation (based on the trees evaluated in STACEY) was carried out using a Java-application ‘speciesDA’ (http://www.indriid.com/software.html).

An input consensus multigene ultrametric tree was the same for “tr2” and STACEY. For this, we combined both sequences in the unified supermatrix via SequenceMatrix v.1.8 (*Vaidya, Lohman & Meier, 2011*), a nucleotide substitution model for each locus was defined in ModelFinder (for the entire 16S sequences and individually for each nucleotide position in the triplet for COI). We deliberately did not delete any sequences with incomplete and missing data, because this approach can greatly reduce the accuracy of the tree reconstruction (*Molloy & Warnow, 2018*). Further analysis was made in the same way as for phylogeny reconstruction, in BEAST2, but with 100M of MCMC generations and sampling every 100k tree. Due to a high uncertainty of the reconstructed tree, we used a final 50% annealing; in our subsequent analysis we used 500 trees from each run. However, the concatenation-based approach (Rokas et al., 2003) is reasonably criticized due to existence of a convergence between restored gene trees and the common species tree (Maddison & Wiens, 1997). To derive a species tree from these different gene trees, we used the multiple fusion technique implemented in ASTRAL-III v.5.6.3 (*Zhang et al., 2018*). No significant differences between the results of two analyses were found in Dendroscope (*Huson & Scornavacca, 2012*), so we used the BI tree for further conclusions.

In order to reduce the impact of a population structure to phylogenetic reconstructions, we previously divided the entire dataset into morphologically defined groups (*excisa*-like, *exigua*-like and *nana*-like) (Tables 1 and 2). Calculations of the nucleotide diversity indices (*Nei & Kumar, 2000*), demographic indicators (mismatch distribution and coalescence modeling for population growth and divergence) and the neutrality tests were performed in dnaSP v. 6.12 (*Rozas et al., 2017*). In order to check the neutrality of the loci and roughly describe possible demographic processes, we carried out the Fs test of neutrality (*Fu, 1997*) and R2 statistics (*Ramos-Onsins & Rozas, 2002*) as the best ways of such analysis (Ramírez-Soriano et al., 2008; Garrigan, Lewontin & Wakeley, 2010). The platform MEGA-X (*Kumar et al., 2018*) was used to calculate genetic distances. We selected “simple”*p*-distances as more preferable for DNA barcoding (*Collins et al., 2012*), as there is no significant difference between uncorrected and corrected substitution models in case of a sufficiently large dataset (*Nei & Kumar, 2000*).

**Table 1:** Genetic diversity of *Alonella* complexes.

**Groups**	**N**	**G+C**	**n**	**S**	**h**	**Hd**	**Pi**	**k**	**Fs**	**R2**
**16S (mitochondrion, rDNA)**										
total 16S	83	0.342	409	184	53	0.985	0.182	70.3	1.039	0.189
*excisa* complex	58	0.346	409	155	36	0.978	0.173	67.8	3.778	0.217
*exigua* complex	17	0.320	409	52	12	0.941	0.042	16.8	0.497	0.158
*nana*	8	0.341	390	125	6	0.893	0.156	61.1	5.132	0.221
**COI (mitochondrion, coding)**										
total COI	78	0.374	626	202	40	0.974	0.166	83.4	5.128	0.204
*excisa* complex	74	0.365	474	184	40	0.965	0.156	74.2	7.040	0.199
*exigua* complex	3	0.373	626	86	3	1	0.037	48.8	5.279	0.357
*nana*	2	0.377	626	0	1	–	–	–	–	–

**Notes.**

N - number of sequences; G+ - guanine-cytosine content; n - total number of sites (excluding sites with gaps / missing data); S - number of segregating (polymorphic) sites; Hd - haplotype diversity; h- number of haplotypes; Pi - nucleotide diversity per site; k - average number of nucleotide differences; Fs - Fu’s neutrality statistic (Fu, 1997); R2- Ramos-Onsins and Rozas R2-statistic (Ramos-Onsins & Rozas, 2002).

## Results

The 16S fragment was successfully amplified and sequenced from most studied individuals. A high rate of unsuccessfully PCRs for COI fragment (27%) is resulted presumably from the presence of sequence mutations at the primer binding sites, as even “universal primers” (Prosser, Martínez-Arce & Elías-Gutiérrez, 2013; Elías-Gutiérrez et al., 2018) did not work properly. The alignment contained 82 newly obtained 16S rRNA sequences (400 bp), 39 original (626 bp) and 39 previously obtained COI sequences deposited in NCBI Genbank or BOLD (Table S1).

Both loci were characterized by a relatively high haplotype and nucleotide diversity and a relatively low G+C portion in the coding COI locus, that, along with previous data (*Kotov et al., 2016*), may be characteristic of the chydorids *in toto*. Results of the neutrality tests for different loci for different groups of populations may indicate multidirectional demographic processes in different lineages and in different loci. Thus, the values of Fs>>0 at R2>0 were characteristic for both loci of the *excisa*-like taxa and may indicate a significant genetic differentiation within this group (with the possibility of splitting/mixing processes in the populations). The *exigua*-like group looks more homogeneous, and the high values of Fs and R2 for COI can be explained by an effect of the small sample size. However, these results demonstrated the need to study in detail the genetic structure of large groups of *Alonella* populations and to resolve the taxonomic uncertainty in these lines.

**Table 2:** Estimates of evolutionary divergence over sequence pairs between *Alonella* complexes. We used uncorrected p-distance (Nei & Kumar, 2000). All ambiguous positions were removed for each sequence pair (pairwise deletion option). On this table COI are located above diagonal, 16S - below diagonal. In the line are within groups p-distance for 16S / COI respectively.

	outgroup	*excisa*	*exigua*	other
outgroup	**out**	0.219	0.209	0.216
*excisa*	0.216	**0.16 / 0.15**	0.202	0.203
*exigua*	0.236	0.197	**0.09 / 0.09**	0.048
*nana*	0.233	0.251	0.229	**0.01 / 0.01**

**Figure 2:**
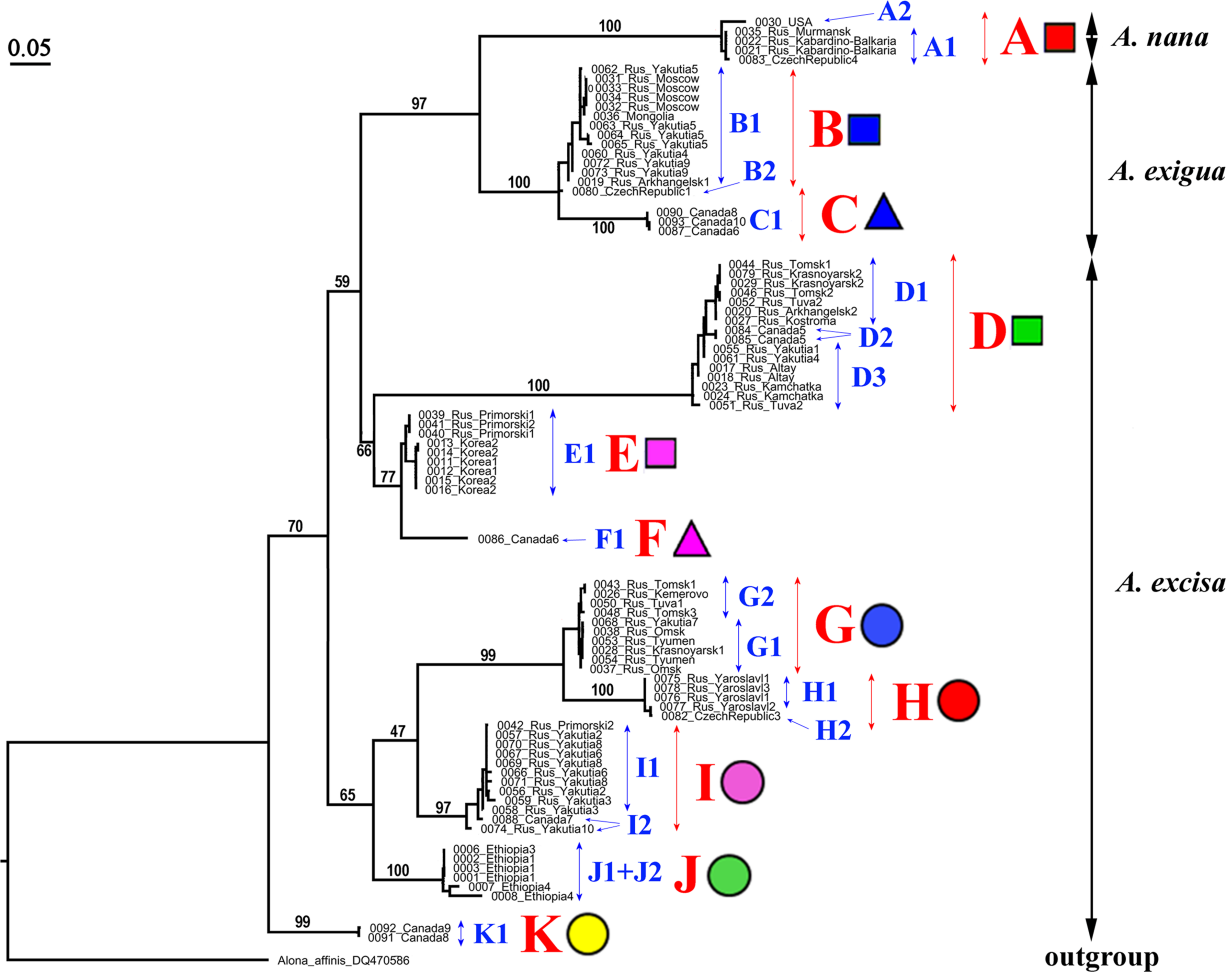
Maximum likelihood tree representing the diversity among phylogroups of *Alonella* based on 16S data. The support values of individual nodes are based on bootstrap-test UFBoot2).

**Figure 3:**
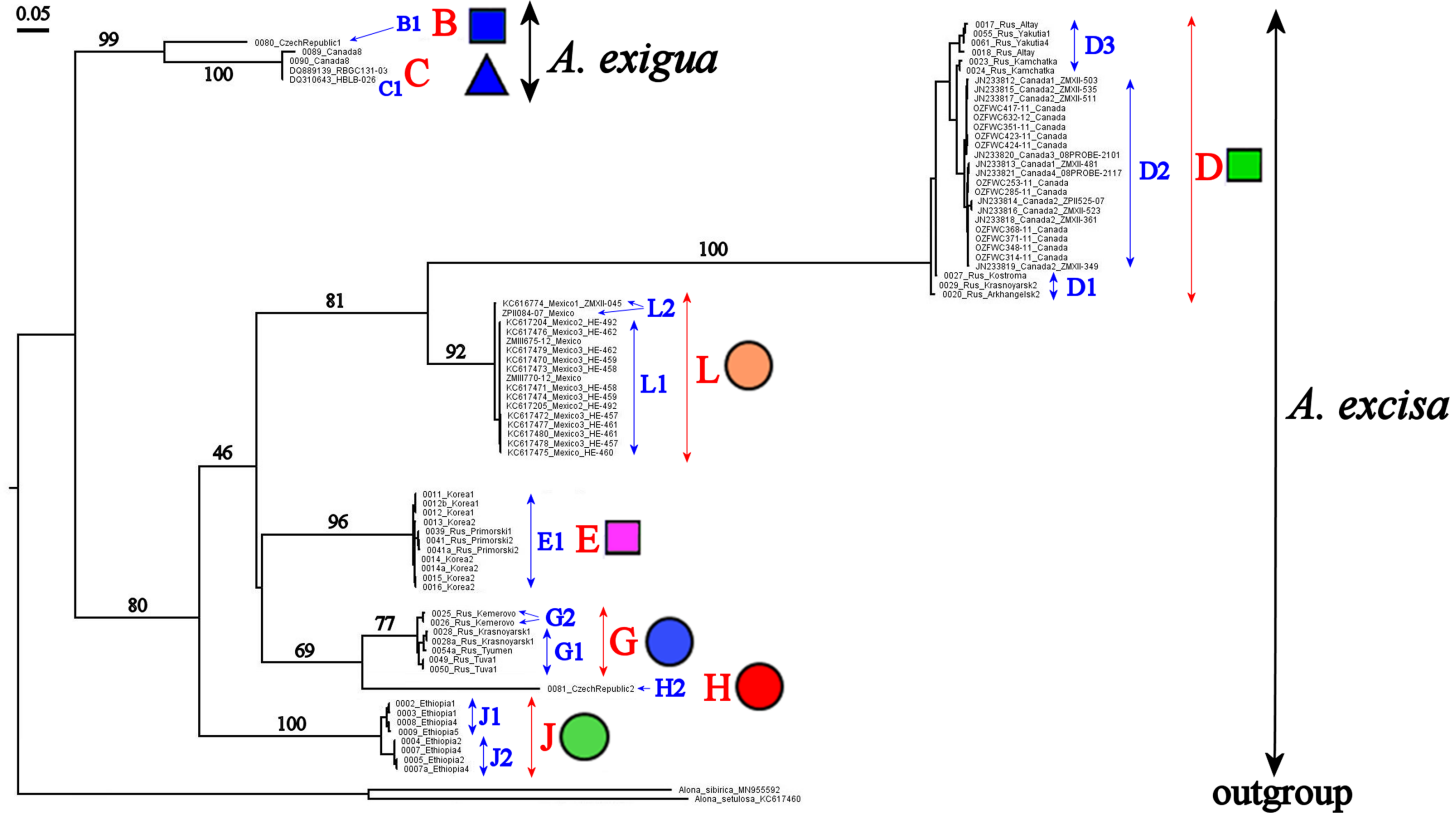
Maximum likelihood tree representing the diversity among phylogroups of *Alonella* based on COI data. The support values of individual nodes are based on bootstrap-test UFBoot2.

Our original sequences together with the GenBank sequences could be assigned to 12 phylogenetically divergent clades, well supported statistically. We numbered all major clades by capital letters from “A” to “L”, the clades A–K are primarily defined based on the variation in the 16S tree (Fig. 2). The clade L is present in the COI tree only (Fig. 3) due to lack of 16S sequences from Mexican populations. In the COI tree, only 8 major clades were represented: B, C, D, E, G, H, J, L (Fig. 3), as we failed to obtain the sequences for clades A, F, I and K. In total, we differentiated a single major clade (A) for *A. nana,* two major clades (B–C) of the *A. exigua* complex and nine major clades (D–L) of the *A. excisa* complex (Figs. 2 and 3).

**Figure 4:**
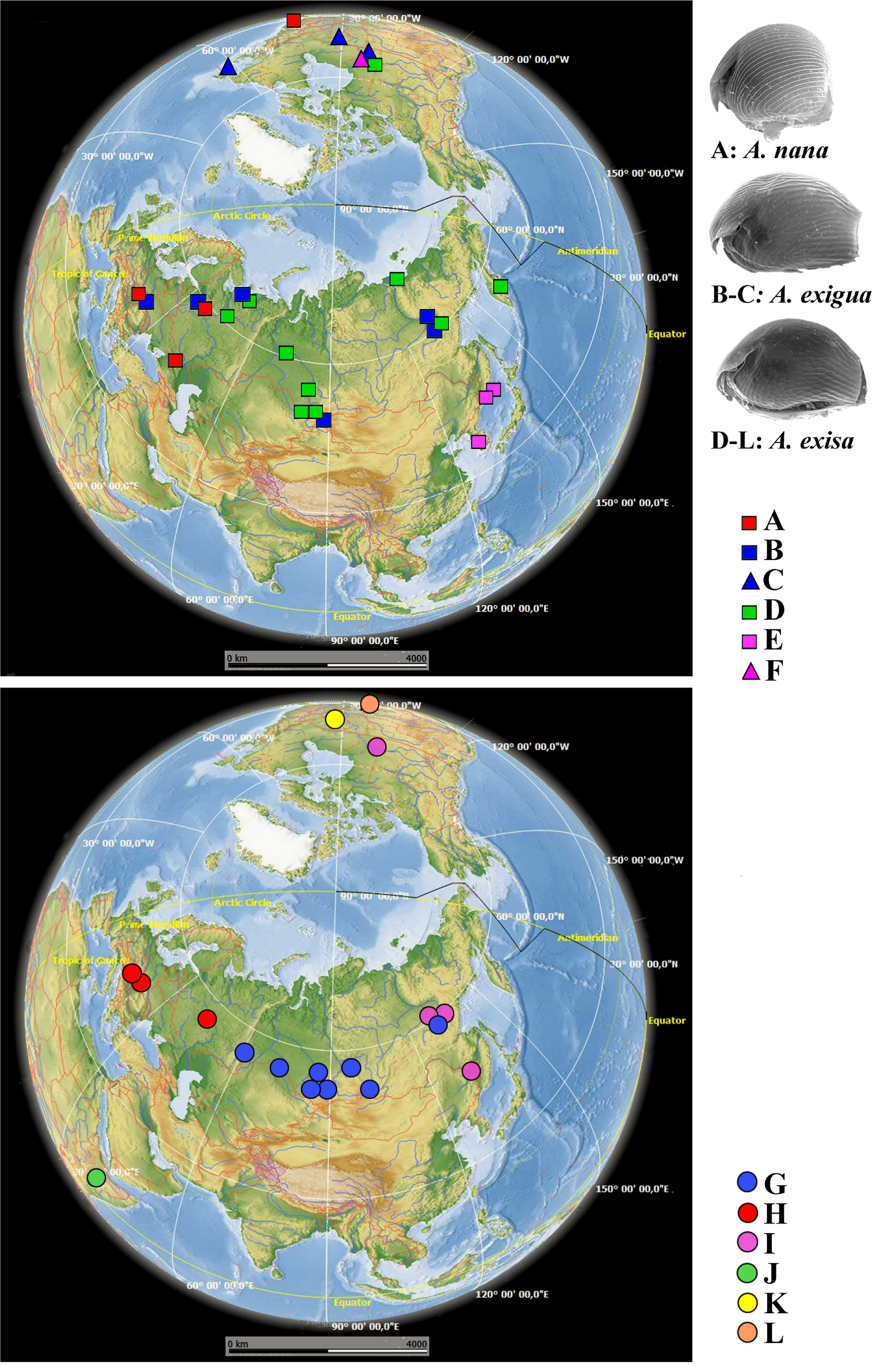
Distribution of major *Alonella* clades (both original and sequences retrieved from NCBI GenBank). The base map was from the open domain plain map available at https://marble.kde.org/.

*Alonella nana* (Fig. 4, upper panel, Table S1). Clade A was represented by two regional subclades: A1 was found in Europe and A2 was found in a single locality in North America.

*A. exigua* complex (Fig. 4, upper panel)*.* Clade B was widely distributed through the northern Palaearctic; sequences from Siberia, Mongolia and European Russia form a subclade B1, while a single Central European sequence formed a separate subclade B2 (The subclade B1 was paraphyletic in the 16S tree) (Fig. 2). The clade C was found in several localities of northern North America forming a single subclade C1 (Newfoundland, Manitoba Ontario).

*A.* cf. *excisa* complex (Fig. 4, upper and bottom panels, Table S1). Clade D was present in the Northern Palaearctic from European Russia to Kamchatka, and also found in Arctic Canada (Fig. 4, upper panel); there were three subclades within the latter: D1 (northern portion of European Russia and Western Siberia), D2 (Manitoba) and D3 (Western Siberia, Eastern Siberia and Kamchatka) (the subclade D3 was paraphyletic in the 16S tree). Clade E was locally distributed in the southern portion of the Russian Far East and South Korea; all sequences formed a single subclade E1. Clade F (with a single subclade F1) was represented by a single sequence from Manitoba (Fig. 4, upper panel). Clade G (Fig. 4, bottom panel) was distributed in Siberia; it was represented by subclade G1 (Western and Eastern Siberia) and G2 (Western Siberia). Clade H (Fig. 4, bottom panel) was exclusively European, and was represented by subclade H1 (Central Europe and European Russia) (this subclade was paraphyletic in the 16S tree) and H2 (Central Europe only). Clade I was widely distributed in the eastern portion of Eastern Siberia, Russian Far East and Manitoba—it formed the subclades I1 (Eastern Siberia and Russian Far East) and I2 (Eastern Siberia and Manitoba) (the latter was paraphyletic in the 16S tree). Clade J was found only in the Ethiopian Bale Mountains, it contained two subclades (J1 and J2) both from this local area. Clade K (with single subclade K1) was found in a single locality in Ontario. Clade L (with single subclade L1) was found in the Yucatan Peninsula (tropical Mexico) (Fig. 4, bottom panel).

Genetic differentiation between the major clades was great (p-distance was 12.1–25.1% for COI, and 10.6–27.2 for 16S, Table S2) as compared to other invertebrates. Such a level of genetic differentiation corresponded to (at least) the species level, even if we applied the highest threshold values of such differences for the invertebrates (Hebert, Ratnasingham & De Waard, 2003; Hebert et al., 2004).

The tanglegrams for mitochondrial genes (Fig. S1 on-line), and species (Fig. S2 on-line) had similar topologies, including the terminal branches. There was strong agreement for the existence of the same major clades in both mitochondrial loci (Fig. S3 on-line). A few discrepancies in tree topologies were detected; however, it was clear that a reliable reconstruction of the tree branching at the high hierarchical level would benefit from a full representation of all clades. Such sampling could not be provided for objective reasons, i.e., part of the data was sourced from previously published studies. Delimitation on the entire general multi-locus tree via different methods, including those based on multi-species coalescence, was illustrated in Fig. S3 on-line. Major clades were recognized as separate units by all algorithms.

## Discussion

### Genetic basis for biodiversity understanding

Based on a logics of the “standard screening threshold of sequence difference (10 × average intraspecific difference”) (*Hebert et al., 2004*), we would have to conclude that each the *excisa*, *exigua* and *nana* is represented by a single polymorphic species. However, the levels of divergence in these complexes are significantly higher than it was previously found in most animals (Ratnasingham & Hebert, 2013; Meier et al., 2006; Huemer et al., 2014; Čandek & Kuntner, 2015). Therefore, an alternative and much more realistic explanation is a high cryptic variability within each studied complex. Possible morphological differences within the aforementioned species complexes must be studied in detail.

**Figure 5:**
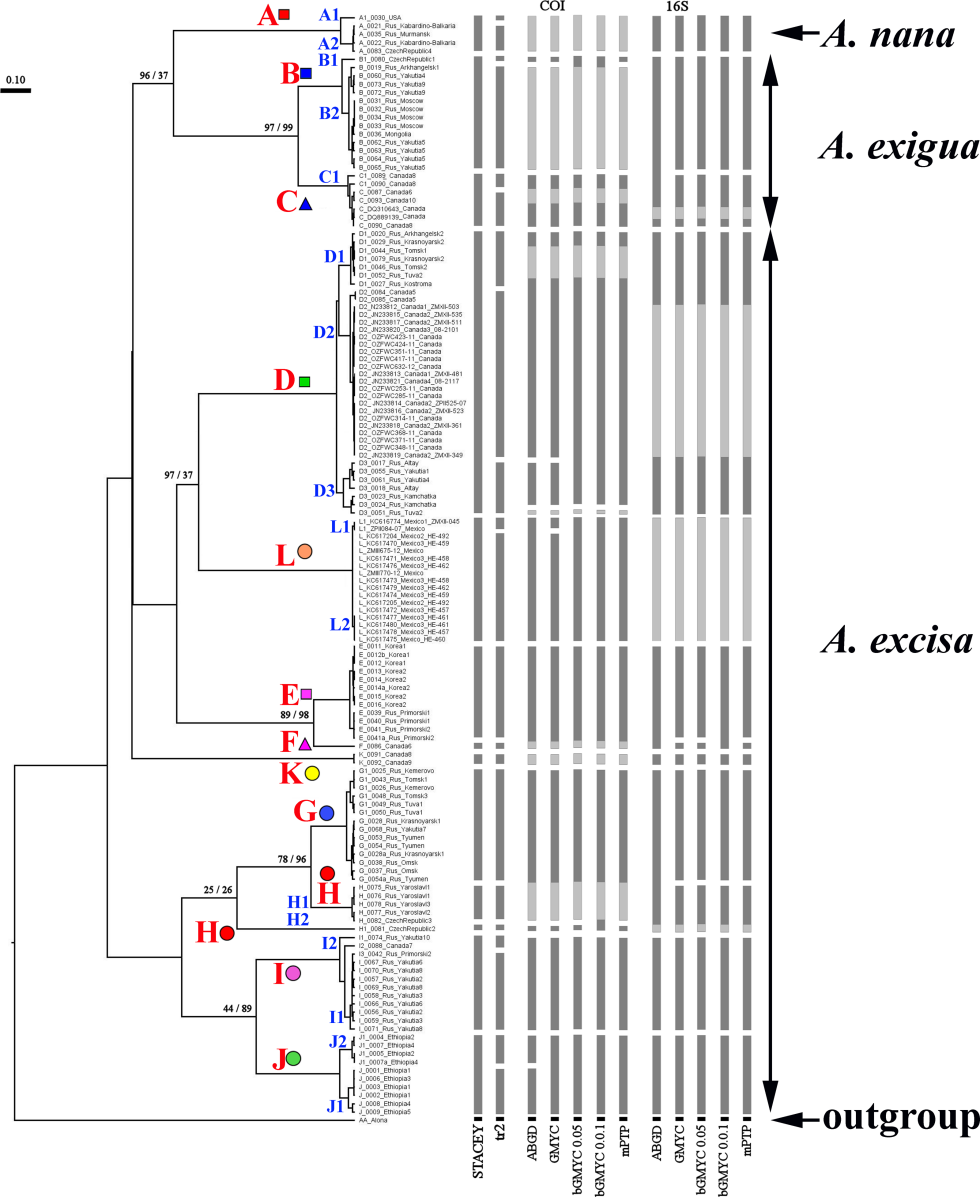
Summary of results of molecular species delimitation via different methods. The BI multi-locus tree is showed. Analyses referring to STACEY and “tr2” are based on multi-locus datasets; for further analytical details, see text. Coloration indicates group membership of specimens; absence of coloration indicates missing data. Node supports are UFboot2 (ML) and posterior probabilities (BI), in percent. Grey color marked absent sequences.

Different delimitation approaches result in different number of distinct units, which may possibly represent species (Fig. 5). The ABGD approach is known by its excessive splitting of the groups with high levels of polymorphism, and as a result, even “good” morphospecies could be easily split into several groups, as it was already shown for *Daphnia magna* (*Bekker et al., 2018*). Theoretically, these problems may have been resolved by using coalescent methods. However, there are obvious “excesses” of such packets as ’splits’ and ‘tr2’, working on the simplest algorithm without an opportunity to correct model parameters based on knowledge about the animal biology. As expected (*Sukumaran & Knowles, 2017*), GMYC models in case of *Alonella* tend to recognize some structured populations as real distinct species (Fig. 5). There are also well-known methodological problems concerning the GMYC (*Maddison & Wiens, 1997; Powell, 2012; Reid & Carstens, 2012*) and PTP (*Zhang et al., 2013; Kapli et al., 2017*) applications. Usually mPTP delimitation is more conservative, only the large groups of populations (Fig. 5) are recognized as species which allows to prevent an excessive splitting (*Tang et al., 2014; Vitecek et al., 2017*). But based on both the analysis of individual trees and species coalescence via several genes, we can state the presence of a complex species structure within the *Alonella* genus. Moreover, the main phylogenetic lineages are supported in all analyses.

As it was shown above, the lack of data on a mitochondrial locus can be compensated *via* data on another locus, it allows us to carry out a reconstruction of the phylogenetic relationships based on the mitogenomes of *Alonella*, to identify main phylogenetic lines, potential OTUs, although they don’t have to be recognized as “biological species” (*Blaxter et al., 2005*).

### Preliminary notes on biogeographic patterns in *Alonella*

Our initial study could not describe fully the biogeographic patterns and phylogeographic scenarios within *Alonella* (i.e., due to obvious sampling limitations), but some preliminary conclusions can be drawn. A separate issue is the possible effect of biological invasions on the formation of modern biogeographic patterns in some clades.

We can classify the patterns of major clades into six groups (Fig. 4):

(1) Trans-Beringian (“Holarctic”) (A, D, I);

(2) Palaearctic (B, G, H) - among which B and G are widely distributed, and H is exclusively European;

(3) Southern Far Eastern (E);

(4) Nearctic, with a pattern unknown to date due to a very limited set of samples from North America (C, F, K);

(5) Mexican Neotropical with unknown real range (L);

(6) Possible endemic Ethiopian (J).

These patterns may indicate a complicated history of dispersion and speciation for *Alonella*. But geographic patterns for the divergent and minor clades of *Alonella* are concordant to those from other cladoceran macro-taxa. A trans-Beringian distribution was observed in some clades of the *Polyphemus pediculus* group (*Xu et al., 2009*) and the *Chydorus sphaericus* group (*Belyaeva & Taylor, 2009; Kotov et al., 2016*). Clade B is widely distributed in the Northern Palaearctic (in our samples, from the Czech Republic to Yakutia Republic), while its sister clade C seems to be restricted to the North America, although presence of both clades in the Far East is also possible. The same situation is observed in the *Moina macrocopa* species group, where *M. macrocopa* s.str. is widely distributed in the Northern Palaearctic, while *M. americana* is restricted to the New World (*Montoliu-Elena, Elías-Gutiérrez & Silva-Briano, 2019*). The clades widely distributed through all the whole northern Palaearctic are also known for the *Polyphemus pediculus* group (*Xu et al., 2009*), *Daphnia curvirostris* group (*Kotov & Taylor, 2019; Kotov et al., 2020*), *D. pulex* group (Crease et al., 2012; Ballinger et al., 2013) and *D. longispina* complex (Yin et al., 2018; Zuykova et al., 2018). Exclusively Nearctic clades are found within many taxa (*Bekker, Kotov & Taylor, 2012; Xu et al., 2011*). Clade E is found in the southern Far East. According to our data, it is distributed from South Korea to Primorski Territory of Russia, but potentially, this taxon may have a wider distribution range as records of thermophilic Oriental taxa in the southern portion of the Russian Far East are not rare (*Kotov, 2016*). But, most probably, this clade belongs to an endemic Far Eastern faunistic complex (Kotov & Taylor, 2019; Kotov, 2016).

An example of the *Alonella* endemism is presumable a specific major clade J from Ethiopian high mountains. In Ethiopia, populations, belonging to the clade J, were detected from the same water bodies where another local endemic, *Daphnia izpodvala*, was found (*Kotov & Taylor, 2010*) and they are never found in the tropical lowlands. At the same time, reliable records of *Alonella* populations from other African countries are very limited. Such records are known from Chad (*Rey & Saint-Jean, 1968*), Fouta Djalon and adjacent mountain areas (*Dumont, 1981*), Cameroon rain forests (*Chiambeng & Dumont, 2005*), Rwenzori mountains (Van Damme & Eggermont, 2011). All these populations have not been studied via genetic methods yet.

Surprisingly, during our study we found some cases of trans-continental geographic ranges in *Alonella* (but only within the Holarctic). Thus, the European subclade A1 is a sister group to A2 from North America (USA, MA) (Fig. 4, upper panel; Fig. S2 on-line). Most likely explanation lies in some past dispersion scenario, with subsequent independent genetic evolution of these newly established populations. Such cases were previously demonstrated for other cladocerans (Taylor & Hebert, 1993; Marková et al., 2007; Millette et al., 2011).

Appearance of the lineages C and F in Canada could be explained as a result of a trans-Beringian transition (Fig. 4, upper panel). Such a transition possibly took place only around 20 thousand years ago, which corresponds well with existence of a massive land bridge between Eurasia and North America, Beringia. The level of genetic differences between them and their sister groups (lineages B and E, respectively) in Eurasia is comparable to that between A1–A2. The presence in Canada of the haplotype from the I2 subclade, close to the Yakutia-Primorsky haplotypes, could be also a consequence of recent anthropogenic introduction from Pacific Asia. Similar patterns are known for other freshwater microcrustaceans (*Ishida & Taylor, 2007*).

The phylogeographic situation is complicated in Canada (Fig. 4, upper and bottom panels; Fig. S2 on-line), where several sympatric main clades and/or subclades were found (D2, K1, F1, C1, I2). The K1 lineage is probably ancestral to the rest of the *Alonella* s.lat. taxa, and its status must be specially checked. The D2 clade is probably a North American phylogenetic lineage of the widespread circumpolar group D, such patterns are already found in *Polyphemus pediculus* (*Xu et al., 2009*). Probably, the subclade C1 and F1 are derived, respectively, from the Eurasian group of populations B and the Far Eastern group E. The comparable genetic distances between these North American and their ancestral groups may be a consequence of their appearance as a result of a trans-Beringian transition. Unfortunately, the Beringian zone is not sampled here, but Beringia apparently has an important role in the *Alonella* biogeographic patterns and needs to be specially studied in the future based on numerous samples.

### Cryptic diversity of *Alonella* across the Northern Hemisphere and short comments on the inter-generic subdivision of *Alonella*

Our study confirms the opinion that the real diversity of the water fleas is several times higher than it is accepted now (*Adamowicz & Purvis, 2005*). This situation is usual for freshwater animals of different groups (*Mills et al., 2017; Schwentner et al., 2020*). We found several possible cryptic species within *A. excisa* and *A. exigua* species complex. To date, characters of the parthenogenetic females have a very limited value for the species discrimination within the *A. excisa* and *A. exigua*. Apparently, incorporation of males to morphological analysis may improve the situation, as it was already shown for some other chydorids (*Belyaeva & Taylor, 2009; Kotov et al., 2016; Garibian et al., 2018*), but, unfortunately, males only sporadically occur in the natural populations of *Alonella*, and, despite our significant efforts, we have no materials with males from some interesting localities, such as Ethiopia.

Before 2010, it was universally accepted that *Alonella* was a monophyletic genus, although the delineation between several chydorid genera (*Alonella*, *Disparalona*, *Pleuroxus* and *Picripleuroxus*) has been intuitive rather than based on accurate diagnostics (*Neretina et al., 2018*). Hudec (2010) subdivided the European taxa of the genus *Alonella* into two subgenera, *Alonella* s.str. and *Nanalonella*. The latter taxon has included a sole species, *A. (N.) nana*, with a globular shape of body, a single minute tooth on posteroventral portion of valve and a very short subquadrangular postabdomen. According to Hudec (2010), *Alonella* s.str. has included in Europe two species: *A. excisa* and *A. exigua*. Both morphospecies are characterized by a somewhat longer, oval body, a somewhat longer, angular postabdomen and posteroventral portion of valve with one or more denticles. Our data suggest that *A. nana* (clade A) is a sister group to *A. exigua* complex (clade B and C) (Fig. 2), in conflict with the subgeneric proposal by Hudec (2010). Variability in the number and shape of these denticles in some chydorids was previously discussed by many authors (*Smirnov, 1996; Kotov, 2013; Neretina & Kotov, 2015; Neretina et al., 2018*), and this feature seems dubious for a reliable discrimination of any subgenera. The same situation concerns the proportions of body and postabdomen. Another strong defect of such classification (*Hudec, 2010*) is that one ignores completely any Non-European *Alonella* taxa.

In fact, morphological differences between the best known *Alonella* species (*Smirnov, 1996*) are less expressed than those between *Pleuroxus* s.l. All attempts to subdivide the latter genus into several genera or subgenera by morphological criteria are controversial due to the mixing of morphological characters in the different taxa (*Smirnov, 1996; Chiambeng & Dumont, 2004*). The taxonomic challenges for *Alonella* and *Pleuroxus* must be resolved with a combination of morphological and genetic data (integrative approach), such studies are known for different microcrustacean groups (*Karanovic & Cooper, 2012; Montoliu-Elena, Elías-Gutiérrez & Silva-Briano, 2019; Ni et al., 2019*). Among the inter-generic subdivisions based on morphological characters carried out in the last two decades for any genera of the subfamily Chydorinae, only attempts to subdivide *Disparalona* s.l. may be considered successful due to the large number of reliable diagnostic features (*Neretina et al., 2018; Neretina et al., 2019*). In general, since the time of Smirnov (1996), morphological taxonomy of Chydorinae is poorly developed. For the latter, the morphological evidence is at its resolution limit, and such studies need to be coordinated with molecular studies.

## Conclusions

Our study reveals a high cryptic diversity within the genus *Alonella* across the Northern Hemisphere. Some of detected main clades have wide ranges across the Old World (and even in the New World), others clades have more restricted ranges, or are likely endemics. Our results could be the basis for subsequent morphological study of *Alonella*, formal description of new taxa and subsequent biogeographical analyses. Thus, biogeographic study is possible for even the smallest of water fleas, as it was also demonstrated for other minute animals, like rotiferans (*Cieplinski, Weisse & Obertegger, 2017; Mills et al., 2017*) or ostracods (*Hiruta et al., 2016*). In this sense, “Little pigeons can carry great messages”.

##  Supplemental Information

10.7717/peerj.10804/supp-1 Supplemental Information 1 Tanglegram for mitochondrial 16S (left) and COI (right) phylogenetic trees Only unique sequences are represented.

10.7717/peerj.10804/supp-2 Supplemental Information 2 Tanglegram for species trees reconstructed by merging in ASTRAL-III (left) and concatenation in BEAST2 (right) with subclades groups Branches support for ASTRAL-III was bootstrap (100 replicas), for BEAST2 was posterior probabilities.

10.7717/peerj.10804/supp-3 Supplemental Information 3 The individual ultrametric BI-trees for 16S (left) and COI (right) loci Node supports are UFboot2 (ML) and posterior probabilities (BI).

10.7717/peerj.10804/supp-4 Supplemental Information 4 Complete list of sequences obtained in the frame of this study and extracted from the GenBank and BOLD with information on specimen ID and locality provided for each individual Clade designations correspond to those in other tables.

10.7717/peerj.10804/supp-5 Supplemental Information 5 Genetic *p.*-distance between *Alonella*’s main clades There are mitochondrial loci above diagonal –COI, below diagonal –16S in the table. In the line are within groups *p*-distance for 16S / COI, respectively.

## Additional Information and Declarations

### Field Study Permissions

The following information was supplied relating to field study approvals (i.e., approving body and any reference numbers): Field collection in public property in Russia does not require permissions. Sampling in the state natural reserves of Russia was conducted with special verbal permission of their heads (O.P. Elizarova, Pinezhsky State Natural Reserve; T.I. Shpilenok, Kronotsky Biosphere Reserve; Y.P. Sushitsky, Khanka Nature Reserve). Sampling in Ethiopian was performed in the frame of the Joint Ethio-Russian Biological Expedition, curated by the Ministry of Environment, Forest and Climate Change of Ethiopia, and does not require special permission. Sampling in Mongolia was performed in the frame of the Joint Russian-Mongolian Complex Biological Expedition, curated by the Ministry of Nature, Environment and Tourism of Mongolia, and does not require especial permission. Samples in South Korea were collected in the frame of cooperation between A.A. Kotov and the National Institute of Biological Resources of Korea and does not require special permission. All the localities in Ethiopia, Mongolia and South Korea belong to public property. They are relatively large lakes, streams, affluents of rivers or roadside ponds.

### DNA Deposition

The following information was supplied regarding the deposition of DNA sequences: DNA described here are accessible at GenBank: MN608113- MN608151 (COI) and MN598677- MN598759 (16S).

### Data Availability

The following information was supplied regarding data availability: Raw data are available in the Supplementary Files.

## References

[ref-1] Adamowicz SJ, Purvis A (2005). How many branchiopod crustacean species are there? Quantifying the components of underestimation. Global Ecology and Biogeography.

[ref-2] Alonso M, Kotov AA (2017). A new species of *Alonella* Sars, 1862 (Crustacea: Cladocera: Chydoridae) from the Ecuadorian Andes. Zootaxa.

[ref-3] Baas Becking LGM (1934). Geobiologie of inleiding tot de Milieukunde.

[ref-4] Ballinger MJ, Bruenn JA, Kotov AA, Taylor DJ (2013). Selectively maintained paleoviruses in Holarctic water fleas reveal an ancient origin for phleboviruses. Virology.

[ref-5] Bekker EI, Karabanov DP, Galimov YR, Haag CR, Neretina TV, Kotov AA (2018). Phylogeography of *Daphnia magna* Straus (Crustacea: Cladocera) in Northern Eurasia: Evidence for a deep longitudinal split between mitochondrial lineages. PLOS ONE.

[ref-6] Bekker EI, Karabanov DP, Galimov YR, Kotov AA (2016). DNA barcoding reveals high cryptic diversity in the North Eurasian *Moina* species (Crustacea: Cladocera). PLOS ONE.

[ref-7] Bekker EI, Kotov AA, Taylor DJ (2012). A revision of the subgenus *Eurycercus (Eurycercus)* Baird, 1843 emend. nov. (Cladocera: Eurycercidae) in the Holarctic with the description of a new species from Alaska. Zootaxa.

[ref-8] Belyaeva M, Taylor DJ (2009). Cryptic species within the *Chydorus sphaericus* species complex (Crustacea: Cladocera) revealed by molecular markers and sexual stage morphology. Molecular Phylogenetics and Evolution.

[ref-9] Blaxter M, Mann J, Chapman T, Thomas F, Whitton C, Floyd R, Abebe E (2005). Defining operational taxonomic units using DNA barcode data. Philosophical Transactions of the Royal Society B: Biological Sciences.

[ref-10] Bolstad WM (2007). Introduction to Bayesian statistics.

[ref-11] Boratyn GM, Camacho C, Cooper PS, Coulouris G, Fong A, Ma N, Madden TL, Matten W, McGinnis SD, Mereshuk Y, Raytselis Y, Sayers EW, Tao T, Ye J, Raytselis Y (2013). BLAST: a more efficient report with usability improvements. Nucleic Acids Research.

[ref-12] Bouckaert R, Vaughan TG, Barido-Sottani J, Duchêne S, Fourment M, Gavryushkina A, Heled J, Jones G, Kühnert D, De Maio N, Matschiner M, Mendes FK, Müller NF, Ogilvie HA, Du Plessis L, Popinga A, Rambaut A, Rasmussen D, Siveroni I, Suchard MA, Wu C-H, Xie D, Zhang C, Stadler T, Drummond AJ (2019). BEAST 2.5: an advanced software platform for Bayesian evolutionary analysis. PLOS Computational Biology.

[ref-13] Čandek K, Kuntner M (2015). DNA barcoding gap: reliable species identification over morphological and geographical scales. Molecular Ecology Resources.

[ref-14] Chiambeng GY, Dumont HJ (2004). The genus *Pleuroxus* Baird, 1843 (Crustacea: Anomopoda: Chydoridae) in Cameroon, Central-West Africa. Annales de Limnologie.

[ref-15] Chiambeng GY, Dumont HJ (2005). The Branchiopoda (Crustacea: Anomopoda, Ctenopoda and Cyclestherida) of the rain forests of Cameroon, West Africa: low abundances, few endemics and a boreal-tropical disjunction. Journal of Biogeography.

[ref-16] Cieplinski A, Weisse T, Obertegger U (2017). High diversity in *Keratella cochlearis* (Rotifera, Monogononta): morphological and genetic evidence. Hydrobiologia.

[ref-17] Collins RA, Boykin LM, Cruickshank RH, Armstrong KF (2012). Barcoding’s next top model: an evaluation of nucleotide substitution models for specimen identification. Methods in Ecology and Evolution.

[ref-18] Costa FO, DeWaard JR, Boutillier J, Ratnasingham S, Dooh RT, Hajibabaei M, Hebert PD (2007). Biological identifications through DNA barcodes: the case of the Crustacea. Canadian Journal of Fisheries and Aquatic Sciences.

[ref-19] Crease TJ, Omilian AR, Costanzo KS, Taylor DJ (2012). Transcontinental phylogeography of the *Daphnia pulex* species complex. PLOS ONE.

[ref-20] Dlouhá S, Thielsch A, Kraus RHS, Seda J, Schwenk K, Petrusek A (2010). Identifying hybridizing taxa within the *Daphnia longispina* species complex: a comparison of genetic methods and phenotypic approaches. Hydrobiologia.

[ref-21] Drummond AJ, Ho SY, Phillips MJ, Rambaut A (2006). Relaxed phylogenetics and dating with confidence. PLOS Biologyl.

[ref-22] Drummond AJ, Suchard MA, Xie D, Rambaut A (2012). Bayesian phylogenetics with BEAUti and the BEAST 1.7. Molecular Biology and Evolution.

[ref-23] Dumont HJ (1981). Cladocera and free-living Copepoda from the Fouta Djalon and adjacent mountain ares in West Africa. Hydrobiologia.

[ref-24] Elías-Gutiérrez M, Valdez-Moreno M, Topan J, Young MR, Cohuo-Colli JA (2018). Improved protocols to accelerate the assembly of DNA barcode reference libraries for freshwater zooplankton. Ecology and Evolution.

[ref-25] Faustova M (2017). Phylogeny, phylogeography and taxonomy of selected members of the family Bosminidae. D. Phil. Thesis.

[ref-26] Faustova M, Sacherová V, Svensson JE, Taylor DJ (2011). Radiation of European *Eubosmina* (Cladocera) from *Bosmina (E.) longispina* - concordance of multipopulation molecular data with paleolimnology. Limnology and Oceanography.

[ref-27] Frey DG (1982). Questions concerning cosmopolitanism in Cladocera. Archiv für Hydrobiologie.

[ref-28] Frey DG, Gore RH, Heck KL (1987). The non-cosmopolitanism of chydorid Cladocera: implications for biogeography and evolution. Crustacean biogeography (Crustacean issues 4).

[ref-29] Fu YX (1997). Statistical tests of neutrality of mutations against population growth, hitchhiking and background selection. Genetics.

[ref-30] Fujisawa T, Aswad A, Barraclough TG (2016). A rapid and scalable method for multilocus species delimitation using Bayesian model comparison and rooted triplets. Systematic Biology.

[ref-31] Fujisawa T, Barraclough TG (2013). Delimiting species using single-locus data and the Generalized Mixed Yule Coalescent approach: a revised method and evaluation on simulated data sets. Systematic Biology.

[ref-32] Garibian PG, Neretina AN, Klimovsky AI, Kotov AA (2018). A new case of West-East differentiation of the freshwater fauna in Northern Eurasia: the *Pleuroxus trigonellus* species group (Crustacea: Cladocera: Chydoridae). Zootaxa.

[ref-33] Garrigan D, Lewontin R, Wakeley J (2010). Measuring the sensitivity of single-locus neutrality tests using a direct perturbation approach. Molecular Biology and Evolution.

[ref-34] Hebert PD, Ratnasingham S, De Waard JR (2003). Barcoding animal life: cytochrome c oxidase subunit 1 divergences among closely related species. Proceedings of the Royal Society of London. Series B: Biological Sciences.

[ref-35] Hebert PD, Stoeckle MY, Zemlak TS, Francis CM (2004). Identification of birds through DNA barcodes. PLOS Biology.

[ref-36] Heled J, Drummond AJ (2009). Bayesian inference of species trees from multilocus data. Molecular Biology and Evolution.

[ref-37] Hiruta SF, Kobayashi N, Katoh T, Kajihara H (2016). Molecular phylogeny of cypridoid freshwater Ostracods (Crustacea: Ostracoda), inferred from 18S and 28S rDNA sequences. Zoological Science.

[ref-38] Hoang DT, Chernomor O, Von Haeseler A, Minh BQ, Vinh LS (2018). UFBoot2: improving the ultrafast bootstrap approximation. Molecular Biology and Evolution.

[ref-39] Hudec I (2010). Anomopoda, Ctenopoda, Haplopoda, Onychopoda (Crustacea: Branchiopoda). Fauna Slovenska III.

[ref-40] Huemer P, Mutanen M, Sefc KM, Hebert PD (2014). Testing DNA barcode performance in 1000 species of European Lepidoptera: large geographic distances have small genetic impacts. PLOS ONE.

[ref-41] Huson DH, Scornavacca C (2012). Dendroscope 3: an interactive tool for rooted phylogenetic trees and networks. Systematic Biology.

[ref-42] Ishida S, Kotov AA, Taylor DJ (2006). A new divergent lineage of *Daphnia* (Cladocera: Anomopoda) and its morphological and genetical differentiation from *Daphnia curvirostris* Eylmann, 1887. Zoological Journal of the Linnean Society.

[ref-43] Ishida S, Taylor DJ (2007). Quaternary diversification in a sexual Holarctic zooplankter, Daphnia galeata. Molecular Ecology.

[ref-44] Jeffery NW, Elías-Gutiérrez M, Adamowicz SJ (2011). Species diversity and phylogeographical affinities of the Branchiopoda (Crustacea) of Churchill, Manitoba, Canada. PLOS ONE.

[ref-45] Jones G (2017). Algorithmic improvements to species delimitation and phylogeny estimation under the multispecies coalescent. Journal of Mathematical Biology.

[ref-46] Kalyaanamoorthy S, Minh BQ, Wong TK, Haeseler Avon, Jermiin LS (2017). ModelFinder: fast model selection for accurate phylogenetic estimates. Nature Methods.

[ref-47] Kapli P, Lutteropp S, Zhang J, Kobert K, Pavlidis P, Stamatakis A, Flouri T (2017). Multi-rate Poisson tree processes for single-locus species delimitation under maximum likelihood and Markov chain Monte Carlo. Bioinformatics.

[ref-48] Karabanov DP, Bekker EI, Shiel RJ, Kotov AA (2018). Invasion of a Holarctic planktonic cladoceran *Daphnia galeata* Sars (Crustacea: Cladocera) in the Lower Lakes of South Australia. Zootaxa.

[ref-49] Karanovic T, Cooper SJ (2012). Explosive radiation of the genus *Schizopera* on a small subterranean island in Western Australia (Copepoda: Harpacticoida): unravelling the cases of cryptic speciation, size differentiation and multiple invasions. Invertebrate Systematics.

[ref-50] Kartavtsev YP (2018). Barcode index number, taxonomic rank and modes of speciation: examples from fish. Mitochondrial DNA Part A.

[ref-51] Katoh K, Standley DM (2016). A simple method to control over-alignment in the MAFFT multiple sequence alignment program. Bioinformatics.

[ref-52] Kotov AA (2013). Morphology and phylogeny of the Anomopoda (Crustacea: Cladocera).

[ref-53] Kotov AA (2016). Faunistic complexes of the Cladocera (Crustacea, Branchiopoda) of Eastern Siberia and the Far East of Russia. Biology Bulletin.

[ref-54] Kotov AA, Garibian PG, Bekker EI, Taylor DJ, Karabanov DP (2020). A new species group from the *Daphnia curvirostris* species complex (Cladocera: Anomopoda) from the eastern Palaearctic: taxonomy, phylogeny and phylogeography. Zoological Journal of the Linnean Society, On-line First.

[ref-55] Kotov AA, Ishida S, Taylor DJ (2009). Revision of the genus *Bosmina* (Cladocera: Bosminidae), based on evidence from male morphological characters and molecular phylogenies. Zoological Journal of the Linnean Society.

[ref-56] Kotov AA, Karabanov DP, Bekker EI, Neretina TV, Taylor DJ (2016). Phylogeography of the *Chydorus sphaericus group* (Cladocera: Chydoridae) in the Northern Palearctic. PLOS ONE.

[ref-57] Kotov AA, Taylor DJ (2010). A new African lineage of the *Daphnia obtusa* group (Cladocera: Daphniidae) disrupts continental vicariance patterns. Journal of Plankton Research.

[ref-58] Kotov AA, Taylor DJ (2019). Contrasting endemism in pond-dwelling cyclic parthenogens: the *Daphnia curvirostris* species group (Crustacea: Cladocera). Scientific Reports.

[ref-59] Kotov AA, Van Damme K, Bekker EI, Siboualipha S, Silva-Briano M, dabache Ortiz AA, Galvándela Rosa R, Sanoamuang L (2013). Cladocera (Crustacea: Branchiopoda) of Vientiane province and municipality, Laos. Journal of Limnology.

[ref-60] Kumar S, Stecher G, Li M, Knyaz C, Tamura K (2018). MEGA X: molecular evolutionary genetics analysis across computing platforms. Molecular Biology and Evolution.

[ref-61] Lampert W (2011). Daphnia: development of a model organism in ecology and evolution. Excellence in Ecology.

[ref-62] Lohse K (2009). Can mtDNA barcodes be used to delimit species? A response to Pons et al. (2006). Systematic Biology.

[ref-63] Maddison WP, Wiens JJ (1997). Gene trees in species trees. Systematic Biology.

[ref-64] Marková S, Dufresne F, Rees DJ, Černý M, Kotlík P (2007). Cryptic intercontinental colonization in water fleas *Daphnia pulicaria* inferred from phylogenetic analysis of mitochondrial DNA variation. Molecular Phylogenetics and Evolution.

[ref-65] Meier R, Shiyang K, Vaidya G, Ng PK (2006). DNA barcoding and taxonomy in Diptera: a tale of high intraspecific variability and low identification success. Systematic Biology.

[ref-66] Millette KL, Xu S, Witt JD, Cristescu ME (2011). Pleistocene-driven diversification in freshwater zooplankton: Genetic patterns of refugial isolation and postglacial recolonization in *Leptodora kindtii* (Crustacea, Cladocera). Limnology and Oceanography.

[ref-67] Mills S, Alcántara-Rodríguez JA, Ciros-Pérez J, Gómez A, Hagiwara A, Galindo KH, Jersabek C, Malekzadeh-Viayeh R, Leasi F, Lee J, Mark Welch D, Papakostas S, Riss S, Segers H, Serra M, Shiel R, Smolak R, Snell T, Stelzer C, Tang CQ, Wallace RL, Fontaneto D, Walsh EJ, Welch DBM (2017). Fifteen species in one: deciphering the *Brachionus plicatilis* species complex (Rotifera, Monogononta) through DNA taxonomy. Hydrobiologia.

[ref-68] Molloy EK, Warnow T (2018). To include or not to include: the impact of gene filtering on species tree estimation methods. Systematic Biology.

[ref-69] Montoliu-Elena L, Elías-Gutiérrez M, Silva-Briano M (2019). Moina macrocopa (Straus, 1820): a species complex of a common Cladocera, highlighted by morphology and DNA barcodes. Limnetica.

[ref-70] Nei M, Kumar S (2000). Molecular evolution and phylogenetics.

[ref-71] Neretina AN, Garibian PG, Romero M, Mondragón DM, Silva-Briano M (2019). A record of *Disparalona hamata* (Birge, 1879) (Cladocera: Chydoridae) in phytotelmata of *Tillandsia aguascalentensis* Gardner, 1984 (Poales: Bromeliaceae). Zootaxa.

[ref-72] Neretina AN, Garibian PG, Sinev AY, Kotov AA (2018). Diversity of the subgenus *Disparalona (Mixopleuroxus)* Hudec, 2010 (Crustacea: Cladocera) in the new and old world. Journal of Natural History.

[ref-73] Neretina AN, Kotov AA (2015). A new species of *Acroperus* Baird, 1843 (Cladocera: Chydoridae) from Africa. Zootaxa.

[ref-74] Nguyen LT, Schmidt HA, Von Haeseler A, Minh BQ (2015). IQ-TREE: a fast and effective stochastic algorithm for estimating maximum-likelihood phylogenies. Molecular Biology and Evolution.

[ref-75] Ni Y, Ma X, Hu W, Blair D, Yin M (2019). New lineages and old species: lineage diversity and regional distribution of *Moina* (Crustacea: Cladocera) in China. Molecular Phylogenetics and Evolution.

[ref-76] Petrusek A, Černy M, Audenaert E (2004). Large intercontinental differentiation of *Moina micrura* (Crustacea: Anomopoda): one less cosmopolitan cladoceran?. Hydrobiologia.

[ref-77] Petrusek A, Hobæk A, Nilssen JP, Skage M, Černý M, Brede N, Schwenk K (2008). A taxonomic reappraisal of the European *Daphnia longispina* complex (Crustacea, Cladocera, Anomopoda). Zoologica Scripta.

[ref-78] Pons J, Barraclough TG, Gomez-Zurita J, Cardoso A, Duran DP, Hazell S, Kamoun S, Sumlin WD, Vogler AP (2006). Sequence-based species delimitation for the DNA taxonomy of undescribed insects. Systematic Biology.

[ref-79] Popova EV, Petrusek A, Kořínek V, Mergeay J, Bekker EI, Karabanov DP, Galimov YR, Neretina TV, Taylor DJ, Kotov AA (2016). Revision of the Old World *Daphnia (Ctenodaphnia) similis* group (Cladocera: Daphniidae). Zootaxa.

[ref-80] Posada D, Buckley TR (2004). Model selection and model averaging in phylogenetics: advantages of Akaike information criterion and Bayesian approaches over likelihood ratio tests. Systematic Biology.

[ref-81] Posada D, Crandall KA (2001). Selecting the best-fit model of nucleotide substitution. Systematic Biology.

[ref-82] Powell JR (2012). Accounting for uncertainty in species delineation during the analysis of environmental DNA sequence data. Methods in Ecology and Evolution.

[ref-83] Prosser S, Martínez-Arce A, Elías-Gutiérrez M (2013). A new set of primers for COI amplification from freshwater microcrustaceans. Molecular Ecology Resources.

[ref-84] Puillandre N, Lambert A, Brouillet S, Achaz G (2012). ABGD, Automatic Barcode Gap Discovery for primary species delimitation. Molecular Ecology.

[ref-85] Rambaut A, Drummond AJ, Xie D, Baele G, Suchard MA (2018). Posterior summarization in Bayesian phylogenetics using Tracer 1.7. Systematic Biology.

[ref-86] Ramírez-Soriano A, Ramos-Onsins SE, Rozas J, Calafell F, Navarro A (2008). Statistical power analysis of neutrality tests under demographic expansions, contractions and bottlenecks with recombination. Genetics.

[ref-87] Ramos-Onsins SE, Rozas J (2002). Statistical properties of new neutrality tests against population growth. Molecular Biology and Evolution.

[ref-88] Ratnasingham S, Hebert PD (2013). A DNA-based registry for all animal species: the Barcode Index Number (BIN) system. PLOS ONE.

[ref-89] Reid NM, Carstens BC (2012). Phylogenetic estimation error can decrease the accuracy of species delimitation: a Bayesian implementation of the general mixed Yule-coalescent model. BMC Evolutionary Biology.

[ref-90] Rey J, Saint-Jean L (1968). Les Cladocères (Crustacés, Branchiopodes) du Tchad. Cahiers ORSTOM. série Serie Hydrobiologie.

[ref-91] Rokas A, Williams BL, King N, Carroll SB (2003). Genome-scale approaches to resolving incongruence in molecular phylogenies. Nature.

[ref-92] Rozas J, Ferrer-Mata A, Sánchez-DelBarrio JC, Guirao-Rico S, Librado P, Ramos-Onsins SE, Sánchez-Gracia A (2017). DnaSP 6: DNA sequence polymorphism analysis of large data sets. Molecular Biology and Evolution.

[ref-93] Sacherová V, Hebert PDN (2003). The evolutionary history of the Chydoridae (Crustacea: Cladocera). Biological Journal of the Linnean Society.

[ref-94] Sarver BA, Pennell MW, Brown JW, Keeble S, Hardwick KM, Sullivan J, Harmon LJ (2019). The choice of tree prior and molecular clock does not substantially affect phylogenetic inferences of diversification rates. PeerJ.

[ref-95] Schwenk K, Sand A, Boersma M, Brehm M, Mader E, Offerhaus D, Spaak P (1998). Genetic markers, genealogies and biogeographic patterns in the Cladocera. Aquatic Ecology.

[ref-96] Schwentner M, Rabet N, Richter S, Giribet G, Padhye S, Cart JF, Rogers DC (2020). Phylogeny and Biogeography of Spinicaudata (Crustacea: Branchiopoda). Zoological Studies.

[ref-97] Scornavacca C, Zickmann F, Huson DH (2011). Tanglegrams for rooted phylogenetic trees and networks. Bioinformatics.

[ref-98] Simon C, Frati F, Beckenbach A, Crespi B, Liu H, Flook P (1994). Evolution, weighting, and phylogenetic utility of mitochondrial gene sequences and a compilation of conserved polymerase chain reaction primers. Annals of the entomological Society of America.

[ref-99] Sinev AY, Karabanov DP, Kotov AA (2020). A new North Eurasian species of the *Alona affinis* complex (Cladocera: Chydoridae). Zootaxa.

[ref-100] Smirnov NN (1971). Chydoridae fauni mira. Fauna SSSR. Rakoobraznie.

[ref-101] Smirnov NN (1996). Cladocera: the Chydorinae and Sayciinae (Chydoridae) of the world. Guides to the identification of the microivertebrates of the Continental Waters of the world. SPB Academic Publishing, Amsterdam.

[ref-102] Smirnov NN, Kotov AA (2010). The morphological radiation of setae in the Cladocera (Crustacea) and their potential for morphogenesis. International Review of Hydrobiology.

[ref-103] Steel M, McKenzie A (2001). Properties of phylogenetic trees generated by Yule-type speciation models. Mathematical Biosciences.

[ref-104] Sukumaran J, Knowles LL (2017). Multispecies coalescent delimits structure, not species. Proceedings of the National Academy of Sciences of the United States of America.

[ref-105] Sweet AD, Boyd BM, Allen JM, Villa SM, Valim MP, Rivera-Parra JL, Wilson RE, Johnson KP (2018). Integrating phylogenomic and population genomic patterns in avian lice provides a more complete picture of parasite evolution. Evolution.

[ref-106] Tang CQ, Humphreys AM, Fontaneto D, Barraclough TG (2014). Effects of phylogenetic reconstruction method on the robustness of species delimitation using single-locus data. Methods in Ecology and Evolution.

[ref-107] Taylor DJ, Hebert PDN (1993). Cryptic intercontinental hybridization in *Daphnia* (Crustacea): the ghost of introductions past. Proceedings of the Royal Society of London, Series B-Biological Sciences.

[ref-108] Trifinopoulos J, Nguyen LT, Von Haeseler A, Minh BQ (2016). W-IQ-TREE: a fast online phylogenetic tool for maximum likelihood analysis. Nucleic Acids Research.

[ref-109] Vaidya G, Lohman DJ, Meier R (2011). SequenceMatrix: concatenation software for the fast assembly of multi-gene datasets with character set and codon information. Cladistics.

[ref-110] Van Damme K, Eggermont H (2011). The Afromontane Cladocera (Crustacea: Branchiopoda) of the Rwenzori (Uganda–DR Congo): taxonomy, ecology and biogeography. Hydrobiologia.

[ref-111] Vitecek S, Kučinić M, Previšić A, Živić I, Stojanović K, Keresztes L, Bálint M, Hoppeler F, Waringer J, Graf W, Pauls SU (2017). Integrative taxonomy by molecular species delimitation: multi-locus data corroborate a new species of Balkan Drusinae micro-endemics. BMC Evolutionary Biology.

[ref-112] Warren DL, Geneva AJ, Lanfear R (2017). RWTY (R We There Yet): an R package for examining convergence of Bayesian phylogenetic analyses. Molecular Biology and Evolution.

[ref-113] Xu L, Han BP, Van Damme K, Vierstraete A, Vanfleteren JR, Dumont HJ (2011). Biogeography and evolution of the Holarctic zooplankton genus *Leptodora* (Crustacea: Branchiopoda: Haplopoda). Journal of Biogeography.

[ref-114] Xu S, Hebert PDN, Kotov AA, Cristescu ME (2009). The noncosmopolitanism paradigm of freshwater zooplankton: insights from the global phylogeography of the predatory cladoceran *Polyphemus pediculus* (Linnaeus, 1761) (Crustacea, Onychopoda). Molecular Ecology.

[ref-115] Yin M, Wan X, Ma X, Gießler S, Petrusek A, Griebel J, Hu W, Wolinska J (2018). Cytonuclear diversity and shared mitochondrial haplotypes among *Daphnia galeata* populations separated by seven thousand kilometres. BMC Evolutionary Biology.

[ref-116] Zhang J, Kapli P, Pavlidis P, Stamatakis A (2013). A general species delimitation method with applications to phylogenetic placements. Bioinformatics.

[ref-117] Zhang C, Rabiee M, Sayyari E, Mirarab S (2018). ASTRAL-III: polynomial time species tree reconstruction from partially resolved gene trees. BMC Bioinformatics.

[ref-118] Zuykova EI, Simonov EP, Bochkarev NA, Abramov SA, Sheveleva NG, Kotov AA (2018). Contrasting phylogeographic patterns and demographic history in closely related species of *Daphnia longispina* group (Crustacea: Cladocera) with focus on North-Eastern Eurasia. PLOS ONE.

