# Supplementary figures and images for: Unexpected mitochondrial lineage diversity within the genus Alonella Sars, 1862 (Crustacea: Cladocera) across the Northern Hemisphere
Source: PeerJ. 2021 Feb 1;9:e10804. doi: 10.7717/peerj.10804 (PMC7860113; doi:10.7717/peerj.10804)

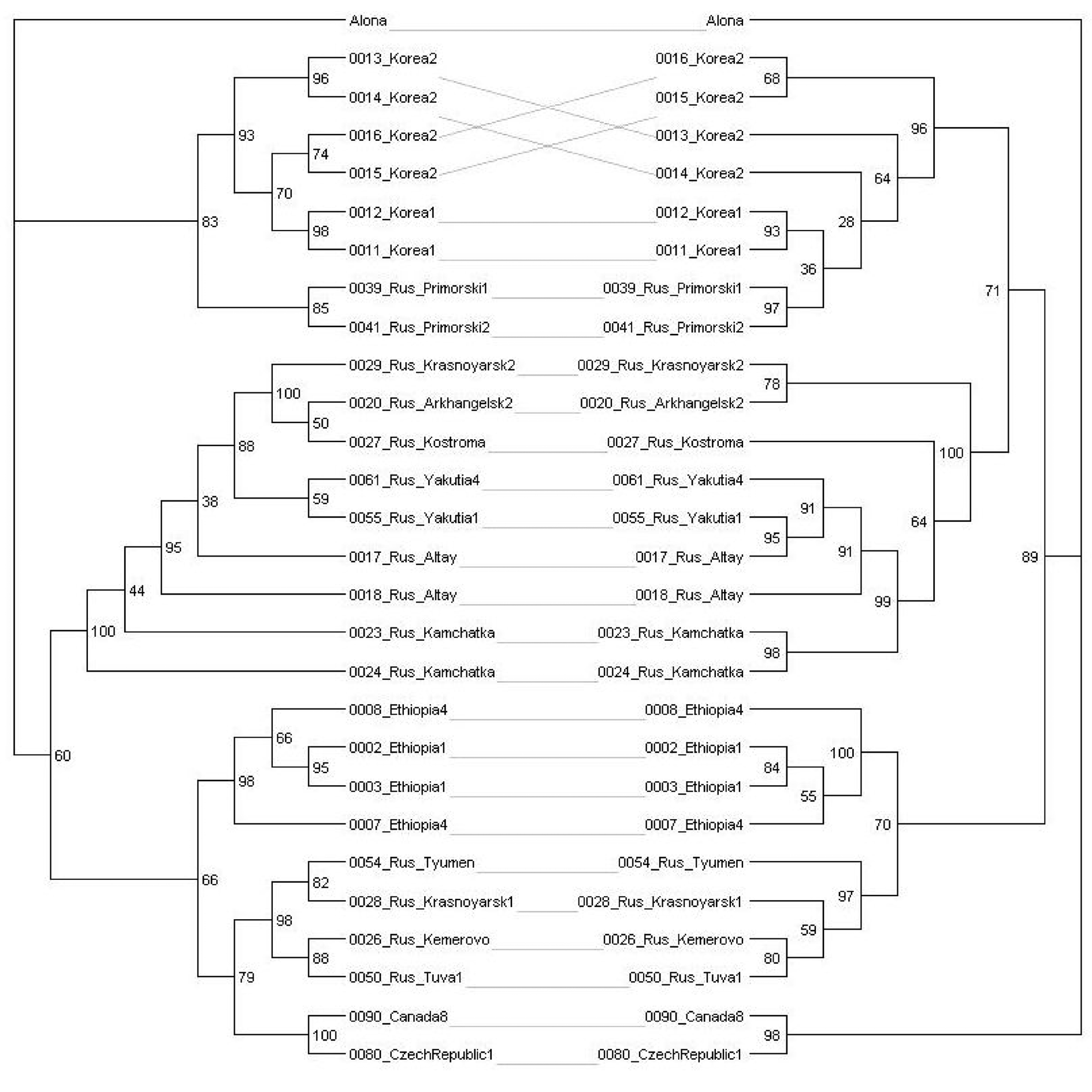

Supplement: Supplemental Information 1 — Only unique sequences are represented. [file peerj-09-10804-s001.png]

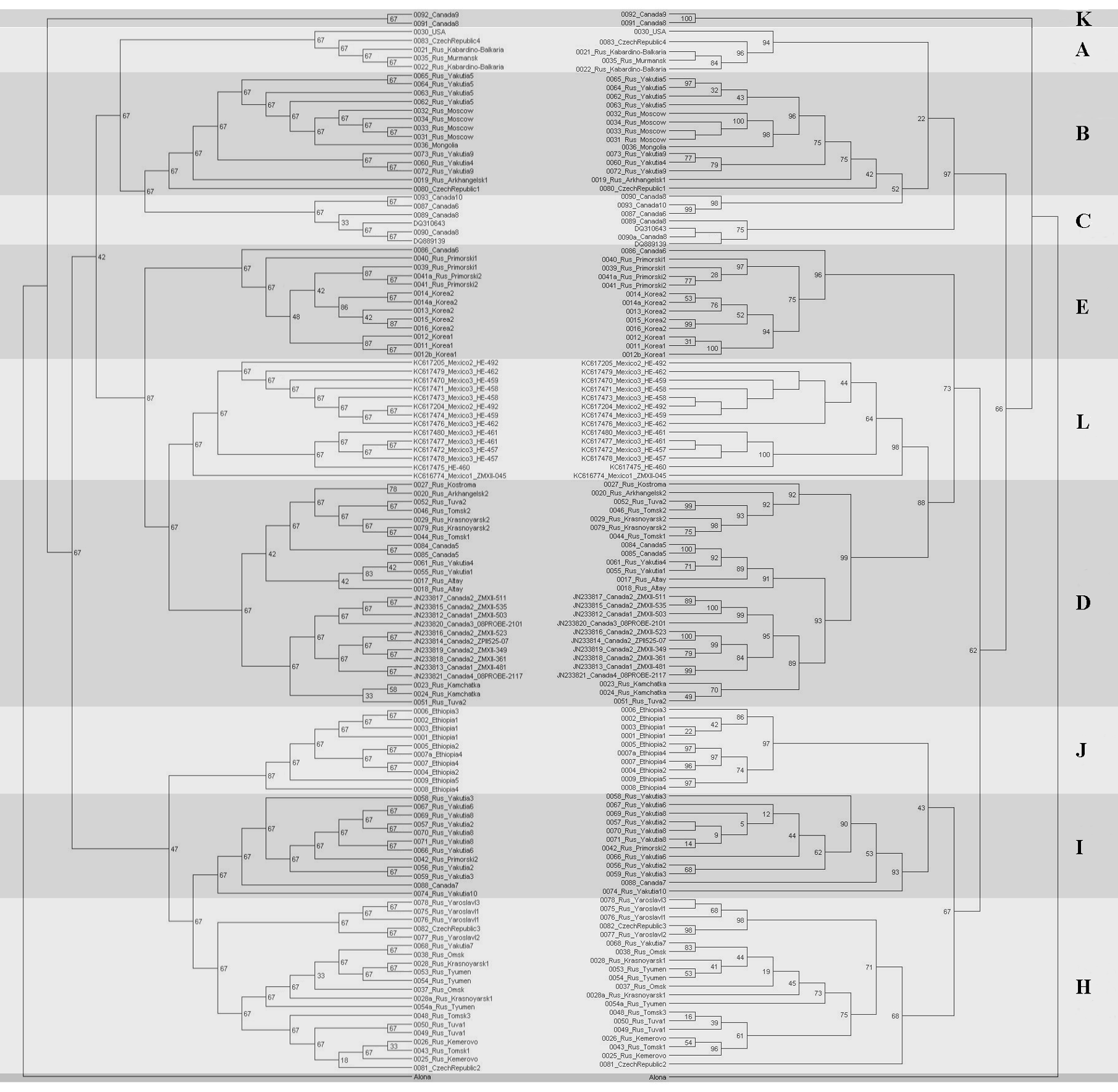

Supplement: Supplemental Information 2 — Branches support for ASTRAL-III was bootstrap (100 replicas), for BEAST2 was posterior probabilities. [file peerj-09-10804-s002.png]

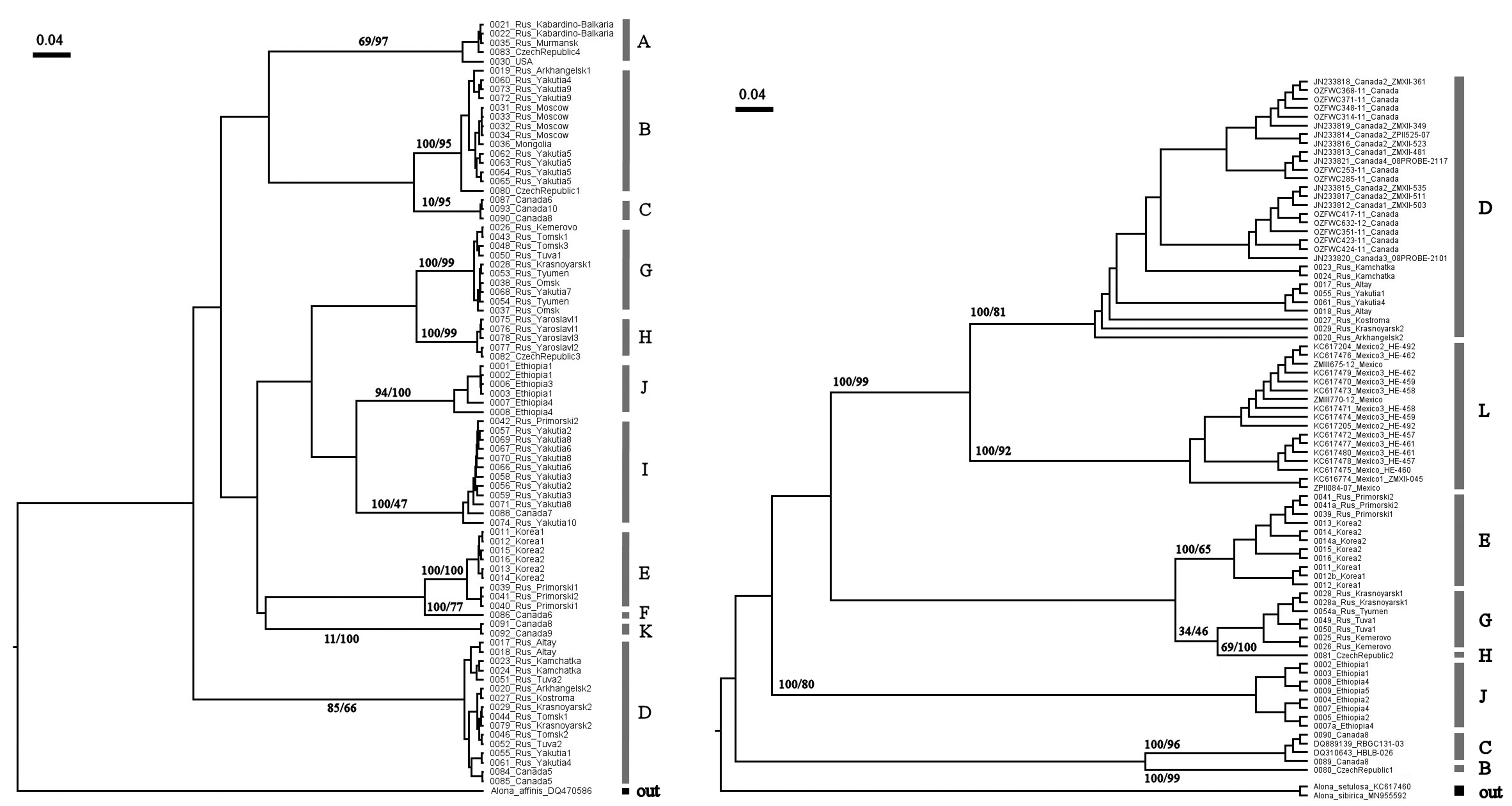

Supplement: Supplemental Information 3 — Node supports are UFboot2 (ML) and posterior probabilities (BI). [file peerj-09-10804-s003.png]
