# Supplementary material for: Unexpected mitochondrial lineage diversity within the genus Alonella Sars, 1862 (Crustacea: Cladocera) across the Northern Hemisphere
Source: PeerJ. 2021 Feb 1;9:e10804. doi: 10.7717/peerj.10804 (PMC7860113; doi:10.7717/peerj.10804)
Supplement: Supplemental Information 5 — There are mitochondrial loci above diagonal –COI, below diagonal –16S in the table. In the line are within groups p-distance for 16S / COI, respectively. [file peerj-09-10804-s005.doc]

|  | outgroup | A | B | C | D | E | F | G | H | I | J | K | L |
| --- | --- | --- | --- | --- | --- | --- | --- | --- | --- | --- | --- | --- | --- |
| outgroup | **out** |  | 19.3 | 21.7 | 23.6 | 21.4 |  | 22.1 | 21.4 |  | 20.9 |  | 19.9 |
| A | 23.8 | **1.6**  **/ -** |  |  |  |  |  |  |  |  |  |  |  |
| B | 25.1 | 19.6 | **1.4**  **/ 2.6** | 12.6 | 21.6 | 18.1 |  | 20.2 | 20.6 |  | 20.8 |  | 16.8 |
| C | 25.2 | 22.6 | 10.6 | **1.1**  **/ 1.4** | 21.5 | 19.0 |  | 21.8 | 22.6 |  | 20.5 |  | 18.8 |
| D | 25.7 | 28.3 | 27.3 | 27.0 | **1.9**  **/ 2.8** | 21.1 |  | 22.7 | 23.0 |  | 25.1 |  | 18.9 |
| E | 21.8 | 22.5 | 18.1 | 19.1 | 20.7 | **1.1**  **/ 0.7** |  | 18.4 | 18.4 |  | 19.2 |  | 14.3 |
| F | 21.3 | 22.8 | 17.4 | 17.8 | 21.7 | 8.3 | **0.1**  **/ -** |  |  |  |  |  |  |
| G | 23.0 | 24.2 | 17.3 | 19.6 | 27.2 | 20.4 | 20.7 | **0.6**  **/ 1.7** | 17.1 |  | 19.2 |  | 17.6 |
| H | 22.2 | 24.9 | 19.5 | 21.5 | 27.1 | 20.1 | 20.9 | 10.5 | **0.5**  **/ 1.2** |  | 22.0 |  | 18.6 |
| I | 21.8 | 25.7 | 20.4 | 23.2 | 26.6 | 15.3 | 18.0 | 17.2 | 19.3 | **1.3**  **/ -** |  |  |  |
| J | 23.1 | 25.3 | 20.5 | 23.2 | 24.7 | 15.9 | 18.0 | 18.0 | 18.5 | 15.1 | **2.2**  **/ 2.3** |  | 18.7 |
| K | 21.4 | 27.2 | 19.7 | 21.1 | 27.2 | 17.3 | 19.6 | 20.1 | 20.1 | 19.7 | 17.1 | **0.2**  **/ -** |  |
| L |  |  |  |  |  |  |  |  |  |  |  |  | **-**  **/ 0.2** |
